# Traumatic Brain Injury Severity in a Network Perspective: A Diffusion MRI Based Connectome Study

**DOI:** 10.1038/s41598-020-65948-4

**Published:** 2020-06-04

**Authors:** Reut Raizman, Ido Tavor, Anat Biegon, Sagi Harnof, Chen Hoffmann, Galia Tsarfaty, Eyal Fruchter, Lucian Tatsa-Laur, Mark Weiser, Abigail Livny

**Affiliations:** 1 Division of Diagnostic Imaging, Sheba Medical Center, Tel-Hashomer, Israel; 2 Sackler Faculty of Medicine, Tel-Aviv University, Tel-Aviv, Israel; 3 Sagol School of neuroscience, Tel-Aviv University, Tel-Aviv, Israel; 4 Department of Radiology and Neurology, Stony Brook University School of Medicine, Stony Brook, NY USA; 5 Department of Neurosurgery, Rabin Medical Center, Belinson, Israel; 6 Department of Mental Health, Israel Defense Forces, Medical Corps, Tel Hashomer, Israel; 7 Department of Psychiatry, Sheba Medical Center, Tel Hashomer, Israel; 8 The Joseph Sagol Neuroscience Center, Sheba Medical Center, Tel Hashomer, Israel

**Keywords:** Problem solving, Diseases of the nervous system, Brain, Trauma

## Abstract

Traumatic brain injury (TBI) is often characterized by alterations in brain connectivity. We explored connectivity alterations from a network perspective, using graph theory, and examined whether injury severity affected structural connectivity and modulated the association between brain connectivity and cognitive deficits post-TBI. We performed diffusion imaging network analysis on chronic TBI patients, with different injury severities and healthy subjects. From both global and local perspectives, we found an effect of injury severity on network strength. In addition, regions which were considered as hubs differed between groups. Further exploration of graph measures in the determined hub regions showed that efficiency of six regions differed between groups. An association between reduced efficiency in the precuneus and nonverbal abstract reasoning deficits (calculated using actual pre-injury scores) was found in the controls but was lost in TBI patients. Our results suggest that disconnection of network hubs led to a less efficient network, which in turn may have contributed to the cognitive impairments manifested in TBI patients. We conclude that injury severity modulates the disruption of network organization, reflecting a “dose response” relationship and emphasize the role of efficiency as an important diagnostic tool to detect subtle brain injury specifically in mild TBI patients.

## Introduction

Traumatic brain injury (TBI) produces disconnection in large-scale brain networks. One of the most common pathologies associated with TBI is diffuse axonal injury (DAI), which is defined as damage to white matter connections^1^. The impact of DAI is usually widespread throughout the brain^2–4^, disrupting the brain’s network integrity. It has been previously proposed that DAI may be a core pathology underlying persistent cognitive impairments after TBI^5,6^. Diffusion tensor imaging (DTI) is particularly sensitive to DAI. This widely used MRI technique maps the diffusion of water molecules and can be used to reconstruct white matter fiber tracts non-invasively^7^. Previous studies investigated the effects of TBI on white matter structures by reconstructing specific well-known tracts such as the corpus callosum and anterior corona radiata^8,9^. However, such a strategy does not address the impact of TBI on overall network integrity.

In recent years, studies have suggested the mathematical field of graph theory as a promising and powerful tool to characterize the organization of complex networks, which can be more sensitive to alterations in white matter. This method has recently been applied to the study of human brain connectivity networks^10–12^. In graph theory, the network consists of a set of “nodes” that represent cortical and sub-cortical anatomical regions, and “edges”, which represent connection properties between these nodes (e.g. white matter fibers), responsible for transferring information in the network. Structural architecture of the brain can be measured in three network organization aspects: integration, the ability to combine information from distant brain regions rapidly; segregation, the ability to carry out neuronal processing within groups of brain regions arranged in modules or clusters; and centrality, the importance of separate nodes within a network. Network organization can also be characterized by hub nodes which are nodes occupying a central position within the network^13^. Hub regions may be particularly vulnerable to the effects of TBI, as diffuse damage to white matter tracts has a differentially large effect on highly connected regions and thus may cause long lasting effects on network function^14,15^.

Graph theory has been adopted as a method of studying network architecture, using both functional and structural connectivity in patients with TBI^16–23^. According to a recent review, only two studies have used strength (the sum of weights connected to a node) to compare between TBI patients and control subjects, none found a significant difference between groups^24^. Mitra *et al*. found that connectivity strength could differentiate mild TBI patients with DAI from healthy controls with an accuracy rate of 68.16%^25^. Higher clustering (the fraction of the node’s neighbors that are also neighbors of each other^26^) of structural networks and lower global efficiency (average inverse shortest path length), which reflects the efficiency of the network in transferring information, were reported^20,21,24^. Also, reduced centrality of hubs was found in regions such as the cingulate cortex, frontal and parietal regions^17,22^. Several studies have found a relationship between connectivity characteristics and cognitive function in healthy subjects^27^ and TBI patients^28^. Fargholm *et al*. showed that graph theory properties such as betweenness and eigenvector centralities were significantly associated with information processing speed, executive function and associative memory in TBI patients^22^. In addition, both Caeyenberghs *et al*. and Kim *et al*. found that reduced global network efficiency corresponded with reduced performance in executive functions in TBI patients^20,23^. Nonetheless, these previous studies examined only patients with moderate to severe TBI and did not include a group of patients with mild brain injury. To the best of our knowledge, only two studies examined network structural connectivity with all injury severity levels, mild to severe TBI patients^29,30^. Both studies examined pediatric TBI patients and not adult TBI patients and did not assess the injury severity effect or the association between injury severity levels and network measures.

Our main aim is to examine the effect of traumatic brain injury severity level on white matter connectivity using graph theory on diffusion MRI based network analysis. We hypothesized that injury severity will modulate the alterations in structural network. In addition, we hypothesized that injury severity will affect the association between structural network topology and cognitive deficits, calculated as the difference between pre-injury and post-injury cognitive scores. The effects of injury severity on white matter connectivity and its association with cognition may further our understanding of TBI outcome.

## Results

Forty-six male participants were included in this study, including 22 adult TBI patients who had an acquired closed head injury at least one year before their enrollment in the study. Of these, 12 had mild (mTBI) and 10 had moderate or severe injury (msTBI). Twenty-four healthy control participants, matched for sex with the TBI survivors, were recruited from the general population. Two of the control subjects were excluded from the analysis due to poor quality of data. No differences were found in time since injury (years) between the mTBI and msTBI patients (mTBI, mean(SD) = 3.673(1.793), range: 23–44; msTBI, mean(SD) = 2.525(1.602), range: 24–36; t = 1.569, p = 0.132). All subjects’ age at assessment ranged from 21 to 35, with small though statistically significant differences between groups (main effect of age at assessment, (F(3,41) = 9.586, p = 0.000). Post-hoc analysis showed that the mTBI group subjects (mean(SD) = 34.916(6.999)) were older than both msTBI (mean(SD) = 28.300(3.773); p = 0.004) and control groups (mean(SD) = 27.09(4.308); p = 0.000). The assumptions for  one-way analysis of variance (ANOVA) were tested using Shapiro-Wilk test for normality and Levene's test for equality of variance.

### Global network measures

**Figure 1:**
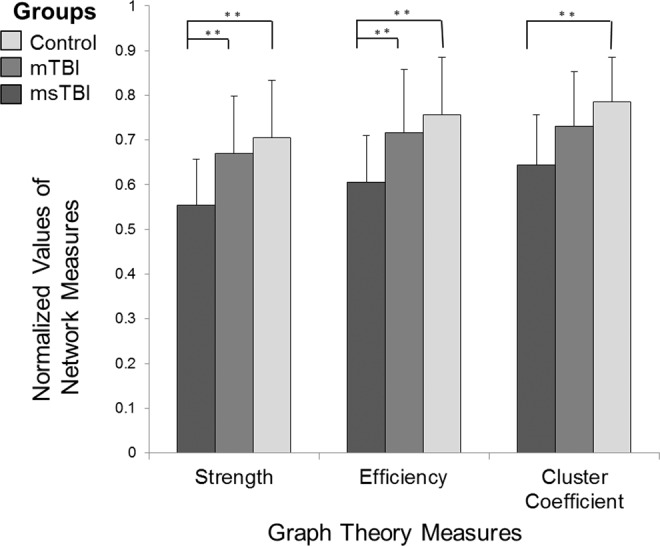
Effects of injury severity on global network measures. Comparison of global network measures among subjects with mTBI (n = 12), msTBI (n = 10) and healthy controls (n = 22) using ANOVA showed a significant difference between groups in strength (p = 0.01), global efficiency (p = 0.012) and clustering coefficient (p = 0.006), with more severe injury associated with a greater decline in network measures. Values are mean ± SD. CPL, Characteristic path length; mTBI, mild traumatic brain injury; msTBI, moderate-severe traumatic brain injury.

The description and mathematical definitions of all measures are presented in Supplementary Table S1. Strength (F_(2,43)_ = 5.169, p = 0.01), global efficiency (F_(2,43)_ = 4.931, p = 0.012) and clustering coefficient (F_(2,43)_ = 5.905, p = 0.006) differed significantly between the injury severity groups (Fig. 1), using the false discovery rate (FDR) correction. The msTBI patients differed from mTBI patients and from healthy controls in strength and efficiency. Also, msTBI patients differed from healthy controls in clustering coefficient. These results indicated a linear effect, in which the more severe the injury was, the more the global measure decreased. We found no correlation between cognitive-change scores and global network measures (change scores’ correlation with global strength (*r* = −0.195, p = 0.384), global efficiency (*r* = −0.13, p = 0.564) and global clustering coefficient (*r* = −0.321, p = 0.145). An additional analysis regarding the global measures, controlled for age, is detailed in the supplementary Table S2a.

We examined the association between injury severity (measured as continuous GCS scores) and network measures in TBI patients. We have found significant correlations between GCS and strength (r = 0.502, p = 0.017) and a marginal effect was found between GCS and efficiency (r = 0.417, p = 0.054). No significant correlation was found between GCS and clustering coefficient (r = 0.340, p = 0.122).

### Network hub regions

**Figure 2:**
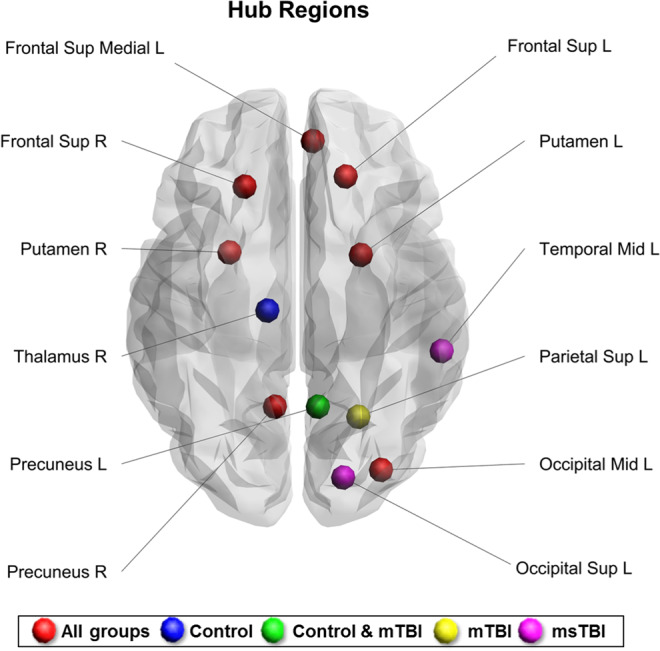
Network hubs across all study groups. R, right; L, left; Mid, middle; Sup, superior; mTBI, mild traumatic brain injury; msTBI, moderate-severe traumatic brain injury.

Hubs were defined as the regions at the top 10% of the ranked betweenness centrality scores (measure of centrality based on shortest paths), representing the brain regions with highest connectivity and centrality. A total of 12 regions were found as hubs overall across all study groups. A detailed table containing the different ranks and order of the top 10% betweenness centrality of each group can be found in supplementary Table S3. Seven network hub nodes were shared between all groups, including: right and left superior frontal gyrus, right and left putamen, right precuneus, left middle occipital gyrus, and left superior medial frontal gyrus. The rank order of five additional hub nodes differed between groups so that the right thalamus, remained a hub solely in the control group; the left precuneus was ranked as a hub only in the control and mTBI groups, but not in the msTBI group; the left superior parietal rank order remained as a hub only in the mTBI group, and the right middle temporal and right superior occipital gyri were hubs only in the msTBI group (Fig. 2).

The three network measures were compared between the groups in each of the 12 regions classified as hubs at least in one study group. Strength significantly differed between groups in bilateral superior frontal and left superior medial frontal cortices. Cluster coefficient significantly differed between groups in the left superior frontal cortex. Control subjects’ strength and cluster coefficient were higher than mTBI and msTBI patients, and the msTBI patients had the lowest values. The efficiency of six regions differed between groups. Of these, bilateral superior frontal, left superior occipital and left middle occipital cortices were more efficient in controls than in mTBI and msTBI patients. The right putamen and left superior parietal cortex presented inconsistent efficiency between groups (Table 1; for post-hoc statistics, please refer to supplementary Table S4).

**Table 1:** Network measures in hub regions.

Network Measures		Strength		Efficiency		Cluster Coefficient	
		mean	SD	mean	SD	mean	SD
Frontal sup L	Control	26243.86	6664.45	0.043	0.008	0.024	0.005
	mTBI	24337.67	6797.60	0.033	0.006	0.021	0.005
	msTBI	16928.50	6436.89	0.034	0.006	0.017	0.004
	p	0.003**		0.000**		0.001**	
Frontal sup R	Control	29299.55	5302.82	0.052	0.012	0.024	0.005
	mTBI	27710.33	6163.02	0.027	0.009	0.023	0.005
	msTBI	21453.80	5685.43	0.025	0.006	0.019	0.007
	p	0.003**		0.000**		0.040*	
Frontal sup medial L	Control	20871.64	4654.06	0.039	0.010	0.025	0.008
	mTBI	19552.92	6189.66	0.033	0.009	0.022	0.005
	msTBI	13153.00	5287.82	0.032	0.006	0.017	0.008
	p	0.002**		0.066		0.022*	
Occipital sup L	Control	16143.32	4365.34	0.027	0.008	0.025	0.007
	mTBI	16563.33	2893.53	0.015	0.008	0.023	0.009
	msTBI	15956.70	4191.42	0.018	0.004	0.026	0.007
	p	0.932		0.000**		0.608	
Occipital mid L	Control	18496.32	6467.73	0.042	0.007	0.021	0.005
	mTBI	16038.92	4914.06	0.039	0.004	0.017	0.006
	msTBI	16308.10	5152.09	0.035	0.004	0.018	0.006
	p	0.416		0.000**		0.108	
Parietal sup L	Control	14296.23	6023.34	0.021	0.009	0.019	0.006
	mTBI	13607.17	4153.52	0.031	0.007	0.016	0.004
	msTBI	12076.90	5132.47	0.028	0.005	0.016	0.005
	p	0.562		0.003**		0.145	
Precuneus L	Control	23719.91	8218.06	0.044	0.015	0.026	0.009
	mTBI	23692.33	7914.93	0.033	0.010	0.022	0.008
	msTBI	19523.70	8711.02	0.035	0.004	0.021	0.006
	p	0.378		0.044		0.235	
Precuneus R	Control	26970.68	7471.84	0.043	0.014	0.028	0.009
	mTBI	24887.42	8190.68	0.037	0.008	0.027	0.011
	msTBI	23395.30	7205.23	0.038	0.009	0.025	0.007
	p	0.444		0.24		0.727	
Putamen L	Control	32325.14	7668.55	0.047	0.006	0.023	0.005
	mTBI	31230.25	8074.44	0.049	0.006	0.021	0.005
	msTBI	26900.90	6459.47	0.051	0.005	0.018	0.007
	p	0.177		0.213		0.048*	
Putamen R	Control	28512.32	6951.38	0.041	0.006	0.018	0.004
	mTBI	28347.50	8210.44	0.046	0.004	0.019	0.005
	msTBI	23589.30	5439.57	0.047	0.005	0.016	0.004
	p	0.169		0.003**		0.342	
Thalamus R	Control	16018.23	4830.37	0.027	0.007	0.017	0.005
	mTBI	17182.17	6444.93	0.024	0.003	0.017	0.004
	msTBI	13045.70	2766.95	0.028	0.010	0.018	0.009
	p	0.149		0.466		0.98	
Temporal mid L	Control	13183.50	2502.74	0.026	0.007	0.019	0.005
	mTBI	13487.50	3263.81	0.020	0.004	0.019	0.008
	msTBI	11656.10	2847.73	0.024	0.007	0.015	0.005
	p	0.268		0.059		0.209	

ANOVA tests showed significant differences between groups in some network measures within the hub regions that were tested (corrected for multiple comparisons, n = 44). SD, standard deviation; R, right; L, left; Mid, middle; Sup, superior; mTBI, mild traumatic brain injury; msTBI, moderate-severe traumatic brain injury.

**Figure 3:**
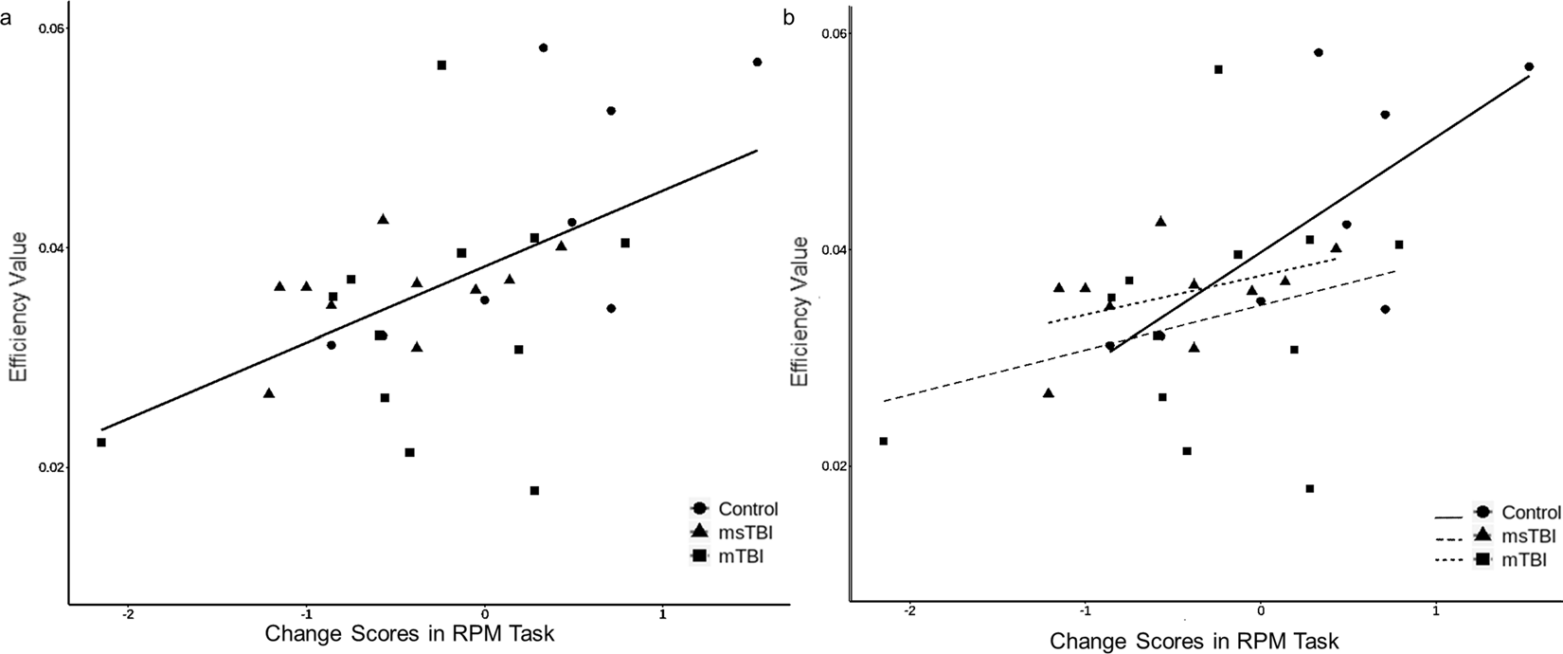
Correlation between efficiency values and cognitive change scores in left precuneus. **a:** correlation between efficiency values and cognitive change scores in left precuneus in all groups together. Pearson correlation test revealed a significant correlation between the RPM change scores and efficiency in the left precuneus (r = 0.53, p = 0.0028, corrected for multiple comparisons), as higher score indicates a more efficient network (n = 30). **b:** Correlation between efficiency values and cognitive change scores in left precuneus in each group separately. Pearson correlation in each study group revealed that the control group maintained a high correlation between efficiency and RPM change scores (*r* = 0.712, p = 0.047, n = 8), while this correlation was lost in both mTBI (*r* = 0.287, p = 0.367, n = 12) and msTBI groups (*r* = 0.457, p = 0.184, n = 10). RPM, Raven progressive matrices; mTBI, mild traumatic brain injury; msTBI, moderate-severe traumatic brain injury.

We examined the correlation between the network measures and Raven’s Progressive Matrices (RPM) task (related to non-verbal abstract reasoning function)^31^ change scores in the five hubs that differed between groups (i.e. right thalamus, left precuneus, left superior parietal, right middle temporal and left superior occipital cortices). A significant correlation was found between efficiency and RPM change scores in the left precuneus (*r* = 0.53, p = 0.003) (Fig. 3a). Examination of the correlations in the left precuneus in each study group separately revealed that this correlation was driven by the control group (n = 8, *r* = 0.712, p = 0.047), and was not significant in the mTBI (n = 12, *r* = 0.287, p = 0.367) and msTBI groups (n = 10, *r* = 0.457, p = 0.184) (Fig. 3b). A significant correlation was also found between cluster coefficient and RPM change scores in the left superior occipital cortex (*r* = −0.445, p = 0.014), however this correlation did not survive FDR correction. No other correlations between network measures of the five hub regions and RPM change scores were found (Table 2).

**Table 2:** Correlation between all network measures and cognitive change scores.

Regions/Network Measure		Strength	Efficiency	Cluster Coefficient
Thalamus R	*r*	−0.123	0.263	0.079
	p	0.517	0.160	0.677
Precuneus L	*r*	−0.248	0.526	−0.113
	p	0.186	0.003**	0.552
Parietal sup L	*r*	0.092	−0.117	−0.055
	p	0.629	0.539	0.773
Temporal mid L	*r*	−0.028	−0.085	0.051
	p	0.884	0.656	0.787
Occipital superior L	*r*	0.004	0.152	−0.445
	p	0.985	0.424	0.014*

Pearson correlation test revealed a significant correlation between the RPM change scores and efficiency in the left precuneus. No other correlations between the RPM change scores and strength, efficiency and cluster coefficient values of the five hub regions were found significant after correction for multiple comparisons. R, right; L, left; Mid, middle; Sup, superior. *p<0.05; **FDR corrected.

### Local network measures

**Figure 4:**
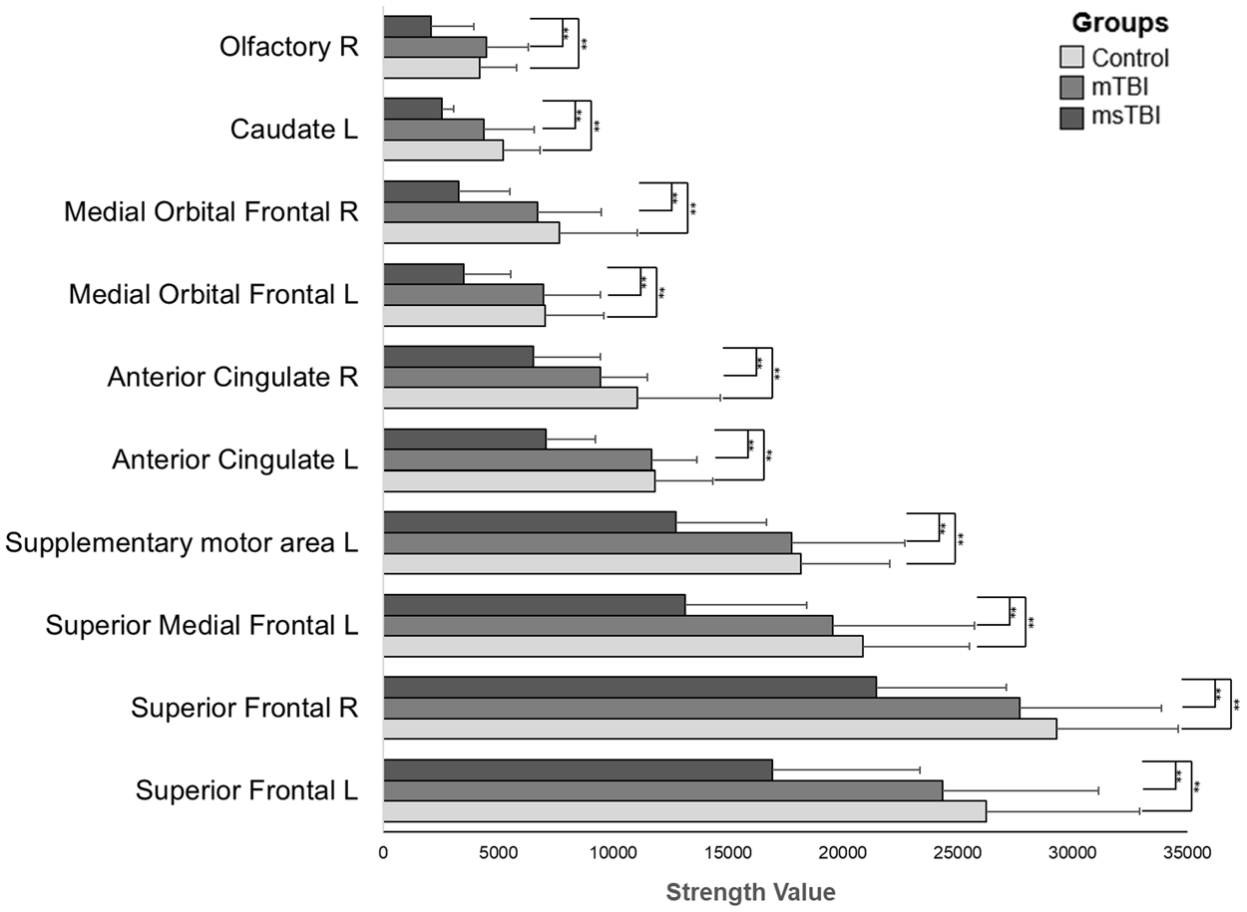
Group differences in local strength in all brain regions. Comparison between mTBI group (n = 12), msTBI group (n = 10) and control group (n = 22) in all brain regions (n = 90) using ANOVA (corrected for multiple comparisons) revealed significant differences between healthy controls and msTBI within 10 regions, with more severe injury associated with a greater decline in network strength. R, right; L, left; mTBI, mild traumatic brain injury; msTBI, moderate-severe traumatic brain injury.

The strength and efficiency measures differed between groups in several regions, after FDR correction for multiple comparisons. The strength of 10 regions was significantly different between the groups, including the right and left superior frontal gyri, left superior medial frontal gyri, left and right anterior cingulum, left and right medial orbital frontal gyri, left caudate, left supplementary motor area and right olfactory cortex. In all regions, the strength value was higher for the control group compared to both TBI groups (Fig. 4). Post-hoc comparisons using LSD (Least Significant Difference) test revealed a significant injury severity effect, whereby msTBI participants exhibited lower strength than control participants and mTBI patients, in all of these regions (for post-hoc statistics, please refer to supplementary Table S5). An additional analysis regarding the local strength, controlled for age is detailed in the supplementary Table (S2b). In addition, local efficiency differed between the groups in 70 regions. However, in this case the results were inconsistent, so that while some regions showed an increase in efficiency, others showed a decrease, with no apparent and coherent relation to the severity of the injury. No injury severity effect was found for local cluster coefficient.

## Discussion

Injury severity was previously associated with changes in white matter integrity^32–34^, as well as gray matter volume decrease^35,36^ and cellular damage^37^. Our study examined the injury severity effect on structural networks connectivity post-TBI, and the association between structural connectivity and cognitive deficits in chronic TBI patients across all TBI severities. To the best of our knowledge, only few structural connectivity studies examined this relationship, and reported no significant association^20,38^, possibly due to lack of pre-injury data. Our findings provide initial evidence that injury severity actually modulates the network organization, which in turn could be responsible for the observed cognitive changes in non-verbal abstract reasoning.

In this study, we have explored the brain network topology post-TBI from a global and local perspective. This wide and comprehensive overview shed light on a “dose-response” effect anchored in all perspectives, by which the network measure value was highest among the control group subjects, intermediate in mTBI and the lowest in the msTBI group. First, using a global perspective, we found significant differences between our study groups, in all three graph theory measures: strength, efficiency and cluster coefficient, suggesting that TBI causes an extensive disruption of integration, segregation and centrality within the brain network. Such disruption suggests that TBI patients’ network connections are relatively less dense than in controls, they have a weaker globally integrated structural brain network and disrupted local neighboring connectivity. This network alteration may result in a limited capacity to combine information across brain regions and lead to a tendency toward a random network and disrupted overall organization. In contrast to our results, some previous studies reported an increase in clustering coefficient^17,21,39^, which implies that brain regions are more connected at a local level across the entire network. However, a recent meta-analysis reported that in adult TBI patients at the chronic phase, normalized clustering coefficient was not significantly higher^24^. In our study, the injury severity was examined using both a categorical measure of severity as well as a continuous one, however we believe that the results of the continuous measure should be taken with caution since results may be influenced by a ceiling effect due to the small distribution of the GCS scores in the mild TBI group, by which, all patients except one presented a GCS score of 15.

Second, looking upon the hub regions, we demonstrate evidence of alterations in certain frontal, occipital, parietal as well as in subcortical hubs. All alterations in the strength and cluster coefficient presented again a dose-response effect. Thus, these network measures are lower in mTBI relative to controls and even lower in the msTBI patients. Finally, using a local perspective, all regions that presented a significant severity effect in the strength graph measure, presented the same consistent dose-response. Therefore, our results consider strength as an essential network measure that can provide notable demonstration of the effect of injury severity.

Network hubs refer to nodes that are highly influential over global brain communication. Damage to their connections has the effect of reducing the robustness and efficiency in transmitting information of the entire network^40^. In the present study, we identified seven hubs shared by all groups, consistent with previous graph-theory studies in healthy control subjects^12,40^. This may indicate that these regions were less affected by brain injury and thus still occupy a central position in the network. Moreover, we identified five hubs which differed between the three groups. The thalamus, which served as a hub only in the control group, is known as a centrally located relay station for transmitting information throughout the brain, and was found previously as a hub region in healthy subjects^40^. Although studies reported the effect of TBI on the thalamus^41–43^, we are the first to describe a decrease in the hub’s rank-order in msTBI patients as well as in mTBI patients, implying vulnerability of the right thalamus’s centrality to the effect of brain injury. The precuneus, which served as a hub in both the control and mTBI groups, has been previously described as a region with the greatest effect on network organization and global network efficiency^44^. In our study, the centrality of the precuneus was lower in the msTBI group. The left superior parietal gyrus was identified as hub only in the mTBI group. This region presented significant changes in cortical thickness in patients with TBI^45^. In contrast to our result, Caeyenberghs and colleagues^20^ identified the superior parietal gyrus as hub for controls and msTBI groups, but no mTBI patients participated in their study. Finally, the right middle temporal and right superior occipital gyri, were identified as hubs only in the msTBI group. To the best of our knowledge, no study has so far reported structural or functional alterations in these regions in TBI patients. Although the network measures in these regions were not associated with cognition (probably due to the small number of control participants with cognitive scores), we postulate that the higher hubs’ rank order of the right middle temporal and right superior occipital gyri may indicate reorganization in msTBI patients.

The examination of the network measures of brain hubs revealed two valuable findings. First, a coherent dose-response effect was displayed in bilateral superior frontal cortices among all three graph network measures, emphasizing the vulnerability of the frontal lobes to the deleterious effects of brain injury^46^. Second, the hubs’ efficiency significantly differed between not only the msTBI and controls, but also between the mTBI and controls, in particular in frontal and occipital regions. The reduced efficiency in these regions may relate to the nature of the coup counter-coup mechanism of injury. Finally, this result underlies the specific importance of the efficiency measure as a sensitive marker for identifying brain injury even in mild severities.

Examination of local strength measure revealed difference between groups in 10 regions. The most affected regions were the cingulum and frontal regions, which are considered as some of the most commonly affected regions by TBI to be identified with DTI studies. Examination of local efficiency revealed conflicting results: 70 regions were significantly different between groups, lacking a consistent pattern of injury severity. The local efficiency measure was discussed in recent studies, presenting the same inconsistency: while some studies found reduced local efficiency^17,23^, others found an increase in this measure^41,47^. This disagreement is likely to be related to the variation in re-organizational effects, in an attempt to compensate for brain injury.

The notion that TBI patients often present cognitive decline is well established^36,45,48,49^. In recent years, it was suggested that graph matrices describing structural brain connectivity could yield a useful description of the underlying cause for cognitive impairments produced by TBI, highlighting the potential of network analysis for better understanding TBI’s outcome. For example, studies have reported that lower global efficiency and centrality values were associated with worse cognitive performance^20,22,23^. Previous studies had suggested that cognitive impairment is particularly produced after TBI when highly connected hub regions are disconnected as a result of axonal injury^16^, and this may be an important mechanism underlying long term cognitive impairment^22^. Our study design had the advantage of having unique access to pre-injury scores of TBI and control subjects from the IDF’s database, enabling us to address the actual size of cognitive deficits following TBI based on actual pre-injury and post-injury cognitive scores rather than estimates. In our study, healthy controls presented a significant association between cognitive change scores over time and the precuneus’ efficiency, but this association was lost in the TBI group. Efficiency in the precuneus could no longer indicate task performance change, suggesting that this disconnection may affect cognition. TBI is regarded as a “disconnection syndrome“^50^, thus one possible explanation for this loss in TBI patients is disconnection of fibers connected to the precuneus, resulting in a longer path to this region thus leading to a less efficient network. The lack of association between the efficiency and cognitive performance may indicate disorganization in the patients’ network, notable even for mild TBI patients. This pattern of disrupted network topology post-TBI can be expressed by several of the re-established connections being more efficient, and others less efficient, which in turn can lead to changes in the association to cognitive performance. This disorganization was also reflected in our local efficiency results, in which we found 70 regions that differed in their efficiency between groups, however no consistent pattern of efficiency was observed.

It is well established in the literature that the precuneus is one of the brain’s most globally connected area^51^ and is regarded as a hub node in the human brain, playing an integrative functional role^52^. Studies have shown that the precuneus is a core part not only in the Default Mode Network (DMN), but also in a variety of high-level cognitive functions^53^, supporting complex cognition and behavior. The precuneus has been also reported to be associated with TBI outcome based on structural and functional neuroimaging findings^54^. Reduced cortical thickness of the precuneus was associated with executive function deficits in TBI patients compared to healthy subjects^55^. The findings derived from our study suggest that the precuneus may play an essential role in the association between efficiency and cognitive deficits in TBI patients.

The current study design has a few limitations. We used a relatively small sample size due to the complexity of recruiting TBI patients who had existing pre-injury cognitive scores. However, we used a statistical procedure to ascertain control for false-discovery rate and minimize the chance for type 1 errors. Despite the relatively low number of subjects, the majority of our findings were statistically significant and supportive of our hypotheses, highlighting the strength of this study. An additional limitation is the relatively heterogeneous sample of patients with respect to localization of impact and mechanism of injury, which could affect our results in respect to the contribution to the complex pattern of network dysfunction. Nonetheless, all patients suffered from a diffuse closed-head injury. In addition, as expected, the three graph theory measures examined in this study, i.e. strength, efficiency and cluster coefficient, are highly cross-correlated with each other and thus the reported global effects of injury severity dose-response on these measures may actually represent the same underlying factor of tract integrity. Nonetheless, the graph theory measures did not present the same effect pattern in the local based analyses. Furthermore, our interpretation of changes in hub regions between the different groups should be taken cautiously since no clear threshold for “hub or not hub” has ever been identified, thus our betweenness-centrality results were based on rank order in the group level of top 10%. Finally, the positive correlation between the RPM change-score and precuneus’ efficiency finding is somewhat surprising considering the small number of subjects in this specific correlation analysis and that the RPM change-score may possibly reflect a noise measurement, this interpretation should be treated cautiously until larger studies will replicate this result.

In conclusion, this is the first study to discover an effect of injury severity on the structural connectome of adults with chronic TBI, using graph theory analysis. Furthermore, a “dose response” effect was reflected in both global and local perspectives. The reduced network efficiency in the precuneus, which was also associated with cognitive deficits, supports the notion that lower connectivity may interfere with successful performance in the abstract reasoning task. This interference may be particularly important in disconnection of network hubs. Hub regions may be more susceptible to brain injury and therefore, damage to them may lead to a less efficient network. In particular, efficiency in hub regions may serve as an important diagnostic tool to detect subtle brain injury in mild TBI victims who do not present overt clinical symptoms.

Our study suggests that graph theoretical analysis of structural connectivity based on DTI may be a sensitive tool for both detecting brain injury and allowing a better understanding of TBI outcome. Future studies should address the relative contribution of specific primary parameters such as FA, neuroinflammation, or levels of neurotransmitters like glutamate and γ-aminobutyric acid to the identifiable changes in network connectivity properties reported in our study. Further investigation should also examine alterations in network topology in the acute phase. Such exploration, together with our results, could lead to advances in current evaluation, diagnosis and prognosis of TBI, and perhaps guide development of new rehabilitative treatments.

## Materials and Methods

### Participants

A total of 46 subjects were enrolled in the study. Twenty-two patients with diffuse closed head injury, including 12 mTBI and 10 msTBI, were recruited from the Neurosurgery Department, Sheba Medical Center. Twenty-four healthy controls with no history of other neurological or psychiatric disorders were recruited from the community. Injury severity was defined using the Glasgow Coma Scale (GCS) within the first 24 hours of hospital admission after injury, and was divided into categories of mild, moderate, and severe injury. GCS scores 14–15 were considered “mild”, while GCS 3–13 were considered “moderate-severe”. Patients were chronic TBI, recruited at least one-year post-injury. The Institutional Ethics Committee of the Chaim Sheba Medical Center, Tel Hashomer approved the study and all patients signed an informed consent. The studies were carried out in accordance with the relevant guidelines and regulations of the ethics committee. The following inclusion criteria were used for TBI participants: 1. Male participants, age 18–45 years old. 2. TBI patients who had suffered a diffuse close head injury at least one year prior to their enrollment to the study (chronic phase). 3. Patients who are fully conscious, able to complete an informed consent and capable of performing cognitive tests. 4. Subjects from all injury severities (GCS scores-3–15), who had received a score equivalent to moderate disability or good recovery on the Glasgow Outcome scale (GOS). 5. Subjects who had undergone pre-military cognitive assessment. 6. No prior TBI requiring hospitalization. Exclusion criteria for both TBI and control groups were: 1. History of other major neurological or psychiatric illness. 2. Use of medication that is likely to substantially affect cognitive performance. 3. Brain surgery post-TBI. 4. Subjects with large lesions on MRI in the time of the study were excluded. 5. Exclusion criteria of MRI: claustrophobia and presence of metal in the body.

### Neuropsychological assessment

All participants underwent a first baseline assessment of cognitive performance as part of the aptitude tests, mandated by the IDF’s pre-military screening draft board, and post-TBI cognitive assessment with analogous version of the same cognitive tests. The IDF’s institutional review board approved the study.

In this study, our cognitive measure was based on non-verbal abstract reasoning function, assessed with the RPM test^31^. The RPM consists of a series of matrices with a missing element that completes a pattern of shapes. The subject’s task is to determine the rule by which the shapes are organized and choose the correct missing shape. In a previous study conducted by our group, we computed a change score between post and pre-injury cognitive scores (post minus pre-injury) for each individual, which represent the differences in performance between the pre-injury and the post-injury scores. This study had shown that the non-verbal abstract reasoning function had declined in both moderate-severe and mild TBI groups (for further details regarding the cognitive change scores, please refer to Livny *et al*. 2016)^36^.

### MRI acquisition

Both TBI patients and healthy control subjects underwent standard T1 and diffusion weighted imaging scans at the Division of Diagnostic Imaging, Sheba Medical Center, acquired on a 3.0 Tesla whole body MRI system (GE, Signa HDxt, version 16 VO2) with an 8-channels head coil.

Spin-echo diffusion weighted echo-planar imaging (EPI) sequences were performed with the following parameters: 40 axial slices with in-plane resolution of 1mm^2^ and slice thickness = 4 mm; TR = 10,000 ms, TE = 91 ms, matrix 128*128 (reconstructed to 256*256) and field of view (FOV) of 256 mm^2^. We acquired 25 diffusion weighted images in isotropically distributed directions, with b = 1000 s/mm^2^ (∆/δ = 33/26 ms) and an additional non-DWI image (b0).

Other structural sequences (T1-weighted and T2 FLAIR) were scanned for clinical diagnosis and lesion evaluation. High-resolution images of the entire brain were acquired for each subject using a standard three-dimensional inversion recovery fast spoiled gradient echo (FSPGR) T1 weighted sequence with the following parameters: repetition time (TR) = 10,000 ms, echo time (TE) = 2.9 ms, flip angle=20°, Inversion time (TI) = 450 ms, field of view (FOV) = 256 mm, voxel size = 1 mm^3^, and matrix size = 256*256. T2-weighted fluid-attenuated inversion recovery (FLAIR) sequence was acquired with the following parameters: TR = 9500 ms, TE = 123 ms, flip angle = 90°, axial slices with FOV = 220 mm, voxel size = 3 mm^3^ with gap of 0.4 mm and matrix size = 64*64.

### Image processing

**Figure 5:**
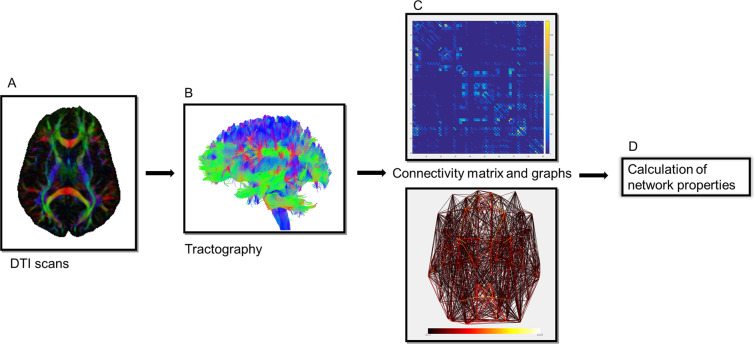
Data processing pipeline of constructing a DTI-based network. (**A**) First, for each subject, a DTI scan was obtained. (**B**) Whole brain tractography was performed using ExploreDTI (see “Materials and methods”). (**C**) The automated anatomical labeling (AAL) template, consisting of 90 brain regions, was then used to create the white matter connectivity matrix, which was filled with number of fibers’ values for the connections between each of the brain regions for every subject, resulting in a 90 × 90 connectivity matrix, which can also be presented as a graph. (**D**) Overall organizational graph theory measures were computed from the resulting brain network. DTI, diffuse tensor imaging.

We constructed a DTI-based network following a data processing pipeline described in Fig. 5, Using ExploreDTI v4.8.6^56^, including: 1. Regularization: images were regularized and resampled (regularization factor of 0.5). 2. Brain extraction: automatic skull stripping and additional manual cleaning to remove areas of remaining skull. 3. Motion correction: diffusion data were corrected for motion and eddy currents distortions using rigid-body transformations to further minimize motion artifacts. 4. DTI calculation: the diffusion tensors were calculated using a non-linear regression procedure^57^.

### Connectivity analyses

For each subject’s dataset, whole brain tractography was performed based on a deterministic streamline fiber tractography approach^58^. Trajectory propagation was terminated if the angle between consecutive steps exceeded 45◦ or if the fractional anisotropy (FA) values were lower than 0.2. The step size was set at 0.75 mm.

Using the Network Analysis Tool embedded in ExploreDTI, nodes and edges were defined. The nodes were characterized using the automated anatomical labeling atlas (AAL)^59^ template, to obtain 90 cortical and subcortical regions (the cerebellar and ventricle regions of interest (ROIs) were excluded). To include information about the magnitude of the connections, which potentially offers greater sensitivity to network alterations caused by injury, weighted connectivity matrices were created. Edges were defined by the number of streamlines connecting each pair of nodes. Since DTI tractography does not differentiate between efferent and afferent fibers, the reconstructed graphs were all undirected. The resulting number of streamlines was converted to square, symmetrical matrices with 90 rows and 90 columns corresponding to all AAL ROIs. Connections with fewer than 10 streamlines were treated as noise and were given a value of zero. The main diagonal was set to zero, excluding self-connections within the region. These symmetrical connectivity matrices were normalized by correcting for the total number of fibers, and then used for further connectivity analyses (Fig. 5C).

Graph theoretical analysis was performed using Brain Connectivity Toolbox (BCT)^60^ in combination with in-house developed MATLAB (v2016b) scripts. The network measures were selected based on previous graph theory studies on TBI populations, and were subdivided into three aspects of network organization, in accordance with the division presented in Van Der Horn 2016: centrality (equivalent to influence), integration and segregation^61^.

All measures used in the current study were based on the work of Rubinov and Sporns^60^ (for a description and mathematical definitions of all measures, refer to Supplementary Table S1). For integration, we computed a measure of global efficiency, defined as the average inverse shortest path length, reflecting the network efficiency in transferring information^62^. The efficiency measure excludes infinitely long paths (i.e. paths between disconnected nodes) from the computations^63^. The measure of network segregation was cluster coefficient, the fraction of the node’s neighbors that are also neighbors of each other^26^. For centrality, we computed a measure of strength, defined as the sum of weights connected to a node. All measures were normalized in order to be examined on the same scale.

Hub regions were defined using ranking of betweenness centrality, expressing the number of all shortest paths (the number of minimal ‘steps’ required for connecting a pair of nodes) in the network that contains a given node. Each node was first ranked separately with a score between 1 and 90; a score of 90 reflecting the highest betweenness centrality. The nodes in the top 10% of the ranked betweenness centrality values scores (9 out of 90 total nodes) were classified as hub nodes, representing the brain regions with highest connectivity and centrality^22^. Accordingly, for each of the participants’ groups we defined hub regions and further performed statistical analyses on these regions.

### Statistical analysis

To investigate the injury severity effect, ANOVA was conducted. Post-hoc analyses were then performed to evaluate pairwise differences among adjusted means. An additional analysis controlled for age was conducted and detailed in the supplementary Table S2. To examine the effect of injury severity on cognition, Pearson’s correlation coefficients were computed between network properties and change scores in the RPM task. To correct for multiple comparisons of different brain regions and connectivity measures, we controlled the FDR using the Benjamini-Hochberg procedure^64^ in all analyses, both global and local, at significance level of 0.05. All analyses were conducted using statistical package for social sciences (SPSS; version 23.0; Armonk, NY: IBM Corp) and in-house developed MATLAB scripts.

## Supplementary information

Supplementary tables.

## Data Availability

The datasets generated and analyzed during the current study will be not publicly available due to confidentiality of the IDF data involved in this study. Any specific request for data could be discussed with the corresponding author who will submit the particular request to the IDF.
