# Supplementary material for: Traumatic Brain Injury Severity in a Network Perspective: A Diffusion MRI Based Connectome Study
Source: Sci Rep. 2020 Jun 4;10:9121. doi: 10.1038/s41598-020-65948-4 (PMC7272462; doi:10.1038/s41598-020-65948-4)
Supplement: Supplementary file 1 — Supplementary tables. [file 41598_2020_65948_MOESM1_ESM.pdf]

Traumatic Brain Injury Severity in a Network Perspective:

A Diffusion MRI Based Connectome Study

Reut Raizman, Ido Tavor, Anat Biegon, Sagi Harnof, Chen Hoffmann, Galia Tsarfaty, Eyal Fruchter, Lucian Tatsa-Laur, Mark Weiser, Abigail Livny.

Measure	Description	Weighted Mathematical Definition
	**Centrality**	
**Strength (degree)**	Number of edges connected to a node.	$S_i = \sum_{j \in N} w_{ij}$
**Betweenness centrality**	Number of all shortest paths (the number of minimal 'steps' required for connecting a pair of nodes) in the network that contains a given node	$BC_z = \sum_{i \in N, j \in N} \frac{\sigma_{ij}(z)}{(\sigma_{ij})}$
	**Integration**	
**Global efficiency**	Mean inverse shortest path length in the network.	$E^w_{glob} = \frac{1}{n} \sum_{i \in N} \frac{\sum_{j,h \in N, j \neq i} (d^w_{ji})^{-1}}{(n-1)}$
	**Segregation**	
**Clustering coefficient**	Fraction of triangles around a node (i.e., fraction of neighbors of node i that are neighbors of each other). This measure reflects the tendency of nodes to cluster together	${C_i}^W = \frac{1}{n} \sum_{i \in N} \frac{2t^w_i}{k_i(k_i - 1)}$
**Local efficiency**	Efficiency of connections between (first-degree) neighbors of a node (i.e., E_{loc_i} reflects the global efficiency of the neighborhoods of node i).	$E^w_{loc} = \frac{1}{2} \sum_{i \in N} \frac{\sum_{j,h \in N, j \neq i} (w_{ij} w_{ih} [d^w_{jh}(N_i)]^{-1})^{1/3}}{k_i(k_i - 1)}$

Supplementary Table S1: A description and mathematical definitions of all measures used in the current study, based on the work of Rubinov and Sporns in 2010.
Edges (i, j) are associated with connection weights w_{ij}. N is the set of all nodes in the network, and n is the number of nodes. a_{ij} is the connection status between i and j. u,v are different modules. d_{ij} is the distance between i and j. k_i is the degree of i. t_i is the number of triangles around i. σ_{ij} is the shortest path between i and j.

a:

Measure	F(2,43)	p
**Strength**	5.117	0.011*
**Global efficiency**	5.040	0.011*
**Clustering coefficient**	5. 575	0. 007*

b:

Regions	Control > msTBI	Control > mTBI	mTBI > msTBI
**Superior Frontal L**	.003*	.988	.037*
**Superior Frontal R**	.003*	.977	.045*
**Superior Medial Frontal L**	.001*	.790	.070
**Supplementary motor area L**	.004*	.932	.082
**Anterior Cingulate L**	.000*	. 993	.001*
**Anterior Cingulate R**	.002*	.650	.143
**Medial Orbital Frontal L**	.002*	.745	.001*
**Medial Orbital Frontal R**	.002*	1	.014*
**Caudate L**	.001*	.950	.018*
**Olfactory R**	.008*	.996	.026*

Supplementary Table S2: A: Global difference between the injury severity groups, using age as covariate and the false discovery rate (FDR) correction. B: Local strength's p-values in all significant regions: post-hoc multiple comparisons using LSD correction with age as a covariate. R, right; L, left; mTBI, mild traumatic brain injury; msTBI, moderate-severe traumatic brain injury. *FDR corrected.

Hub's Rank	Control	mTBI	msTBI
**1**	Putamen R	Hippocampus R	Putamen R
**2**	Hippocampus R	Superior Frontal Orbital L	Superior Frontal Orbital L
**3**	Thalamus R	Middle Occipital L	Middle Temporal L
**4**	Superior Parietal R	Putamen R	Putamen L
**5**	Superior Frontal Orbital L	Superior Parietal L	Hippocampus R
**6**	Putamen L	Superior Parietal R	Superior Frontal L
**7**	Hippocampus L	Hippocampus L	Superior Parietal R
**8**	Middle Occipital L	Thalamus R	Insula R
**9**	Superior Frontal R	Putamen L	Middle Occipital L

Supplementary Table S3: hub's rank in each group, using betweenness centrality; R, right; L, left.

Region/Network Measures	Strength		Efficiency		Cluster Coefficient	
	Groups	p	Groups	p	Groups	p
**Superior Frontal L**	Control > msTBI	.001*	Control > msTBI	.001*	Control > msTBI	.000*
	Control > mTBI	.429	Control > mTBI	.000*	Control > mTBI	.046*
	mTBI > msTBI	.013*	mTBI < msTBI	.738	mTBI > msTBI	.078
**Superior Frontal R**	Control > msTBI	.001*	Control > msTBI	.000*	ANOVA was not significant	
	Control > mTBI	.436	Control > mTBI	.000*		
	mTBI > msTBI	.013*	mTBI > msTBI	.714		
**Superior Frontal medial L**	Control > msTBI	.000*	Control > msTBI	.045*	Control > msTBI	.006*
	Control < mTBI	.488	Control > mTBI	.071	Control > mTBI	.187
	mTBI > msTBI	.007*	mTBI > msTBI	.772	mTBI > msTBI	.159
**Superior Occipital L**	ANOVA was not significant		Control > msTBI	.000*	ANOVA was not significant	
			Control > mTBI	.000*		
			mTBI > msTBI	.314		
**Middle Occipital L**	ANOVA was not significant		Control > msTBI	.003*	ANOVA was not significant	
			Control > mTBI	.000*		
			mTBI < msTBI	.378		
**Superior Parietal L**	ANOVA was not significant		Control < msTBI	.035*	ANOVA was not significant	
			Control < mTBI	.001		
			mTBI > msTBI	.329		
**Putamen L**	ANOVA was not significant		ANOVA was not significant		Control > msTBI	.015*
					Control > mTBI	.262
					mTBI > msTBI	.198
**Putamen R**	ANOVA was not significant		Control < msTBI	.006*	ANOVA was not significant	
			Control < mTBI	.005*		
			mTBI < msTBI	.922		

Supplementary Table S4: Network measures in hubs: post-hoc multiple comparisons using LSD correction.
ANOVA tests showed significant differences between groups in some network measures within the hub regions that were tested (corrected for multiple comparisons, n=44). R, right; L, left; mTBI, mild traumatic brain injury; msTBI, moderate-severe traumatic brain injury. *FDR corrected.

Regions	Control > msTBI	Control > mTBI	mTBI > msTBI
**Superior Frontal L**	.001*	.429	.034*
**Superior Frontal R**	.001*	.436	.013*
**Superior Medial Frontal L**	.000*	.488	.007*
**Supplementary motor area L**	.001*	.800	.007*
**Anterior Cingulate L**	.000*	.886	.000*
**Anterior Cingulate R**	.001*	.165	.035*
**Medial Orbital Frontal L**	.000*	.936	.002*
**Medial Orbital Frontal R**	.000*	.391	.011*
**Caudate L**	.000*	.172	.012*
**Olfactory R**	.002*	.656	.003*

Supplementary Table S5: Local strength's p-values in all significant regions: post-hoc multiple comparisons using LSD correction. R, right; L, left; mTBI, mild traumatic brain injury; msTBI, moderate-severe traumatic brain injury. *FDR corrected.
